# Does treating proximal cavities in primary molars non-restoratively affect intra-arch space and alignment of successor teeth negatively? A 4-year longitudinal study

**DOI:** 10.7717/peerj.14008

**Published:** 2022-10-03

**Authors:** Rafael T. Gomide, Jo E. Frencken, Jorge Faber, Anne Marie Kuijpers-Jagtman

**Affiliations:** 1 Department of Pediatric Dentistry, Universidade de Brasília, Brasília, Distrito Federal, Brazil; 2 Department of Dentistry – Oral function and Prosthetic Dentistry, Radboud University Nijmegen, Nijmegen, Netherlands; 3 Brasília, Brazil; 4 Department of Orthodontics, University Medical Center Groningen, University of Groningen, Groningen, Netherlands; 5 Department of Orthodontics and Dentofacial Orthopedics, School of Dental Medicine/Medical Faculty, University of Berne, Berne, Switzerland; 6 Faculty of Dentistry, Universitas of Indonesia, Jakarta, Indonesia

**Keywords:** Proximal cavity, Dental caries, ART, UCT, Malocclusion, D+E space, Mixed dentition, Tooth migration, Prospective study, Permanent dentition

## Abstract

**Background:**

Removing plaque with toothbrush and toothpaste from proximal cavities in primary molars without restoring them follows sound cariological principles. But does this treatment affect space for and alignment of their permanent successors negatively?

**Hypothesis:**

There is no difference in impaction and displacement of the premolars, as well as in the D+E space in quadrants with three different statuses of the proximal surface of primary molars over a 4-year period.

**Methods:**

A total of 936 quadrants (466 maxillary and 470 mandibular quadrants) in 233 children were assessed. Treatment of cavities in the proximal surfaces of the primary molars consisted of amalgam and ART restorations using high-viscosity glass-ionomer cement, and cleaning of open large- and medium-sized cavities with toothbrush and toothpaste (UCT) under supervision for 220 days per year over 3 years. Dental casts were made at baseline, and after two, three, and 4 years. The D+E spaces were measured digitally. Status of the proximal surface of the primary molars was assessed by two calibrated examiners, and quadrants were grouped into normal anatomy, defective restoration, and proximal cavity. ANCOVA, ANOVA and LSD tests were applied.

**Results:**

There was a statistically significant difference between groups (*p* **<=** 0.001) and between evaluation times (*p* < 0.001), for the D+E space in both the maxilla and mandible. A sex difference related to the D+E space in the maxilla was found (*p* = 0.007). For boys, quadrants in the maxilla of the group ‘proximal cavity’ showed a significant shorter D+E space when compared to quadrants of the group ‘normal anatomy’ at the 3- and 4-year evaluation time. For girls the difference between the two groups was only present at the 3-year evaluation time. There was no significant difference between the D+E space in quadrants with defective restorations and those with normal anatomy in the mandible and in the maxilla. Displacement and impaction of the premolars showed no significant difference between groups.

**Conclusion:**

Primary molars with open proximal cavities that are cleaned with toothbrush and toothpaste do not result in displacement and impaction of the successor teeth, neither do primary molars with defective restorations in proximal tooth surfaces.

## Introduction

Untreated caries in primary teeth is very frequently observed, as shown in a report on the most prevalent oral conditions globally (Marcenes et al., 2013). Out of 291 oral conditions, open cavities in primary teeth ranked number 10, with 621 million children affected worldwide during the period 1990–2010.

Dental caries, in principle, is a preventable disease. The fact that millions of children live with open cavities is a burden for the children and their families, the dental and medical profession, and nations around the world. Factors that contribute to this undesirable situation include lack of attention to oral diseases in global health policies and failure tackling their known social and commercial determinants; emphasis on traditional restorative care as the predominant treatment; and treatment costs that exceed available resources in many low- and middle-income countries (Peres et al., 2019; Watt et al., 2019).

Preventing carious lesion formation can be achieved by regular disturbance of the biofilm on enamel and dentine. This cariological principle is the basis for caries prevention and cavity treatment (Kidd & Fejerskov, 2004). The common treatment of cavitated teeth is restoring them. However, it is known that traditional restorative care frequently causes dental anxiety; leads to low restoration-survival percentages, particularly in approximal surfaces; and is expensive. Another, more causal, cavity treatment concerns the removal of biofilm from open tooth cavities in primary molars (Mijan et al., 2014). This kind of treatment has become the basis of cavity treatment in the guidelines of pediatric dental care in the Netherlands, with effect from January 2021 (KIMO, 2020).

The effectiveness of removing the biofilm from open cavities in primary canines and molars was investigated in a three-arm quasi-randomized clinical trial. The experimental group was termed Ultra-Conservative Treatment (UCT) and treatment consisted of cleaning accessible medium- and large-sized cavities with toothbrush and fluoride containing toothpaste and restoring small cavities according to the Atraumatic Restorative Treatment (ART) method using high-viscosity glass-ionomer cement (HVGIC) (Mijan et al., 2014). UCT was compared with amalgam and ART/HVGIC, with symptomless exfoliation of primary canines and molars as the outcome variable. After 3.5 years, no differences were observed between the three types of treatment, indicating that cavities in primary teeth left open and cleaned do not necessarily lead to a higher percentage of premature tooth loss than restored cavities (Mijan et al., 2014). This result questions the need for traditional restorative care as the predominant treatment for managing cavities in primary teeth.

UCT offers advantages as its cavity-cleaning component is a causal disease treatment, but it relies on good cooperation between the child/parents and the professional in achieving this. It is child-friendly and improves oral hygiene in permanent teeth (Hilgert et al., 2017). However, these positive aspects are nullified if proximal cavities that are left open and cleaned eventually lead to orthodontic problems in the permanent dentition.

It has been suggested that extensive cavities in proximal surfaces of primary teeth are associated with a decrease of space in the primary molar region, known as the D+E space (Northway & Wainright, 1980; Gomide et al., 2020b). Decrease of the D+E space may have orthodontic implications (Stahl & Grabowski, 2004), since it tends to reduce the available space for the eruption of the permanent canine and the premolars, which may affect alignment of the successor teeth and/or cause tooth impaction (Northway, 2000). If this event proves likely, the loss of space due to extensive cavities may also have important financial implications through an increase in the cost of providing orthodontic treatment for millions of patients globally.

By the same token, if cavitated lesions in proximal surfaces of primary molars do not lead to an increase in malalignment, more treatment options for proximal carious lesions in the primary molars can be considered. Instead of prioritizing treatment methods that restore the contact point, one might consider, for example, restorative strategies that prioritize health, cost reduction, and patient comfort, all included in the UCT method.

Limited information is available on the effect on intra-arch distances when cavitated primary molars are subjected to the ultra-conservative treatment protocol (UCT) A pilot investigation into the effect of proximal grinding of carious lesions of primary molars on the available space showed no clinically relevant space loss, but in this study the intention was to leave at least a point of contact between the ground primary molar and the adjacent tooth (Ingers et al., 1982). At the same time, we were not able to find studies that focused on the association of restoration failure or proximal cavities with intra-arch distances. In the current study, we tested the null-hypothesis that there is no difference in the D+E space as well as in impaction and displacement of the premolars in quadrants with three different statuses of the proximal surface of primary molars over a 4-year period. The aim of the study was to assess the association between different statuses of the proximal surface of the primary molars and the D+E space, and displacement and impaction of the premolars over a 4-year follow-up period.

## Materials and Methods

### Study design

The Research Ethics Committee of the University of Brasília Medical School approved the study (ref number 081/2008). The trial was registered in the Netherlands Trial Registry (NTR; ref number 1699). The study design and restorative treatment methods that were used in the present investigation have been described in detail elsewhere (Mijan et al., 2015). A brief description is presented below.

In the original trial, the exfoliation and survival percentages of primary molars submitted to three restorative treatment strategies were assessed over a 3.5-year period (Mijan et al., 2014; Mijan et al., 2015). The treatment protocols consisted of (1) conventional restorative treatment using amalgam (CRT); (2) ART; and (3) UCT. The UCT protocol consisted of restoring small dentine cavities using the ART method and increasing access to medium-sized cavities with a hatchet, with the child then brushing medium and large cavities under supervision during school hours (220 days/year for 3 years) for biofilm removal using toothbrush and fluoride-containing toothpaste.

The investigation was set up as a quasi-randomized, controlled clinical trial with a parallel group design. The subjects were nested in an oral health epidemiological survey that was carried out on 6- and 7-year-old children attending six public primary schools in a socially deprived suburban area of Brasilia, Brazil, in April and May 2009 (Mijan et al., 2014). Only healthy children, having at least two cavitated dentine carious lesions in primary molars without pain and pulp involvement, and whose parents/guardians had signed the informed consent were considered eligible for inclusion in the study (Mijan et al., 2014).

### Dental cast analysis and outcome measures

Data were collected as previously described (Gomide et al., 2020a). Immediately after completion of the restorative treatment, alginate impressions (Avagel®, Dentsply, Petrópolis, Brazil) of both jaws were made with autoclavable mouth trays (Morelli®, Sorocaba, Brazil) and a wax bite was made. The impressions were poured in plaster (Asfer®, São Caetano, Brazil) within 1 h. The same procedure was followed after 2 (T2), 3 (T3), and 4 years (T4).

Measurements were carried out digitally on standardized occlusal pictures of the plaster casts. The method to obtain the occlusal pictures have been described elsewhere (Gomide et al., 2020b).

#### Intra-arch variables

Measurements were performed on the occlusal pictures of the upper and the lower dental arch using a morphometric program (Digimizer v. 4.2, MedCalc Software, Ostend, Belgium) by one calibrated examiner (RG). The examiner performed the measurements under standardized conditions for about 10 weeks between 8 and 10 am every day at the same location under the same lighting conditions. The D+E spaces were measured at baseline (T0), and after 2 (T2), 3 (T3), and 4 years (T4). Tooth displacement and impaction of the premolars were assessed on the final cast (T4). To assess the intra-observer reliability, 29 dental casts were re-assessed after a period of 2 months.

The following variables were measured for both arches and for each quadrant separately.

*D+E space*: measured from the most mesial point of the first permanent molar to the most distal point of the primary canine in each dental arch (in millimetres) in both quadrants. If the first permanent molar was absent, the most distal point of the second primary molar or premolar was considered. If the primary canine was absent, the most mesial point of the first primary molar or premolar was considered. The distance was disregarded if all teeth were missing (Gomide et al., 2020a).

*Displacement of the premolars*: The distance was measured between anatomical contact points when the teeth deviated from the line of the arch (Richmond et al., 1992). The largest contact point displacement in each quadrant was recorded. Displacements between contact points of rotated teeth were disregarded (Richmond et al., 1992). Assessment took place at the final evaluation point (T4) only.

*Impaction of premolars*: This involves the space measured between two teeth situated at both sides of an unerupted tooth. A premolar was considered impacted when the distance between the two teeth was equal to or shorter than 4 mm (Richmond et al., 1992). Tooth impaction was assessed at the final evaluation point (T4) only.

#### Status of the proximal tooth surface

The presence of cavitated dentine carious lesions at the distal surface of the canine and the proximal surfaces of primary molars was determined from digital standardized occlusal pictures of the dental casts at baseline and at all evaluation points with a magnification between two and eight times (Samsung Galaxy Note 10.1, Suwon, South Korea) by two calibrated examinors (RG, JF) independently. Differences were discussed until consensus was reached. To assess the inter-observer reliability, 248 photographs were re-assessed by both observers after a period of 2 weeks.

**Figure 1:**
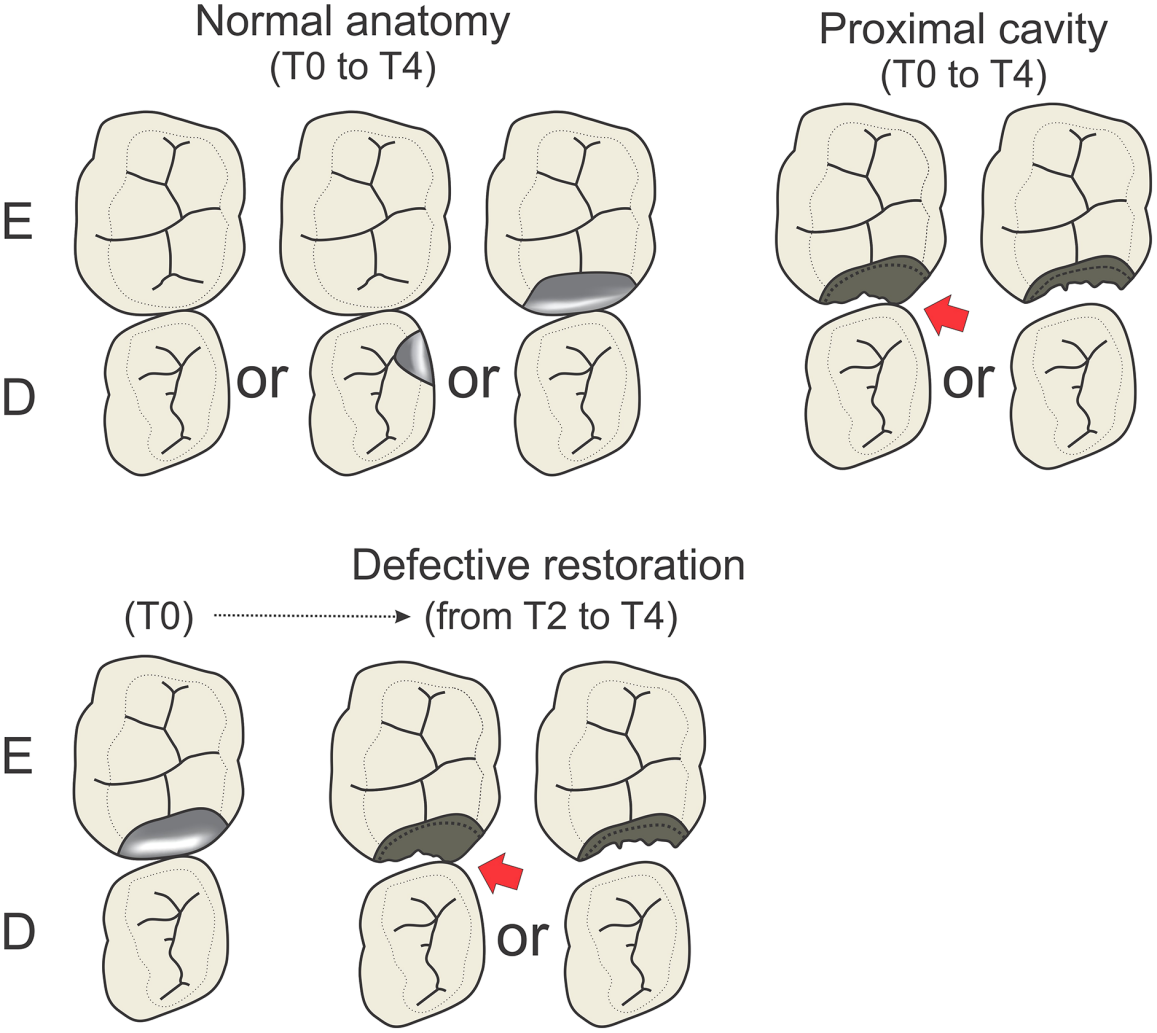
Illustration showing different *“status of the proximal surface”*, grouped into normal anatomy, defective restoration and proximal cavity.

The quadrants were grouped according to the condition of the proximal tooth surface between the primary molars (Fig. 1). The canines were not considered.

– *Normal anatomy:* quadrants with sound proximal surfaces of the primary molars or with intact (proximal) restorations in primary molars in contact with an adjacent tooth, measured at baseline (T0) and all other evaluation points (T2, T3, T4).
– *Defective restorations:* quadrants with restored proximal surfaces at baseline that were assessed as defective at T2 and had lost contact with the adjacent tooth surface, showing an open proximal cavity with and without remnants of restorative material. The defective restorations were not re-restored between T2 and T4.
– *Proximal cavity:* open proximal cavity with or without contact with the adjacent tooth surface at all evaluation points. The cavities had been cleaned with toothbrush and toothpaste under supervision during schooldays for 3 years.

### Statistical analyses

The sample size for the present sub-study had been calculated for the controlled clinical trial. In brief, the sample size was set at 88 individuals for each of the three treatment groups (CRT, ART, UCT) on the basis of a power of 80%, a significance level of 0.05, a 10% correction for dependency on treatments within a child, and an 8% estimated annual loss of children (Mijan et al., 2014).

Kappa statistics was applied to determine the interobserver reproducibility for assessing the status of the proximal tooth surfaces. The intra-examiner reliability for the D+E spaces and tooth displacement was expressed as Pearson’s correlation coefficients. Paired sample t-tests were applied to identify systematic differences between the first and second measurement.

Descriptive statistics were calculated, and outliers received the same value as the mean for the dependent variable (D+E space or tooth displacement). Mean and 95% confidence interval of the D+E spaces in both arches were calculated at each evaluation point. ANOVA’s at T0, T2, T3 and T4 contrasted differences between groups (normal anatomy, defective restoration, and proximal cavity). Post-hoc LSD tests were used when necessary. A mixed model ANCOVA analysis was performed to assess the effect of age (covariate), sex, evaluation time (T0, T2, T3 and T4) and group (normal anatomy, defective restorative and proximal cavity) on the D+E space. A mixed model ANCOVA analysis was performed to assess the effect of age (covariate), sex and group (normal anatomy, defective restorative and proximal cavity) on tooth displacement at the end of the study (T4). Post-hoc LSD tests were used when necessary. Chi-square tests were used to assess the association of group with tooth impaction at the end of the study (T4). The alpha level was set at 5%.

## RESULTS

### Sample distribution

**Figure 2:**
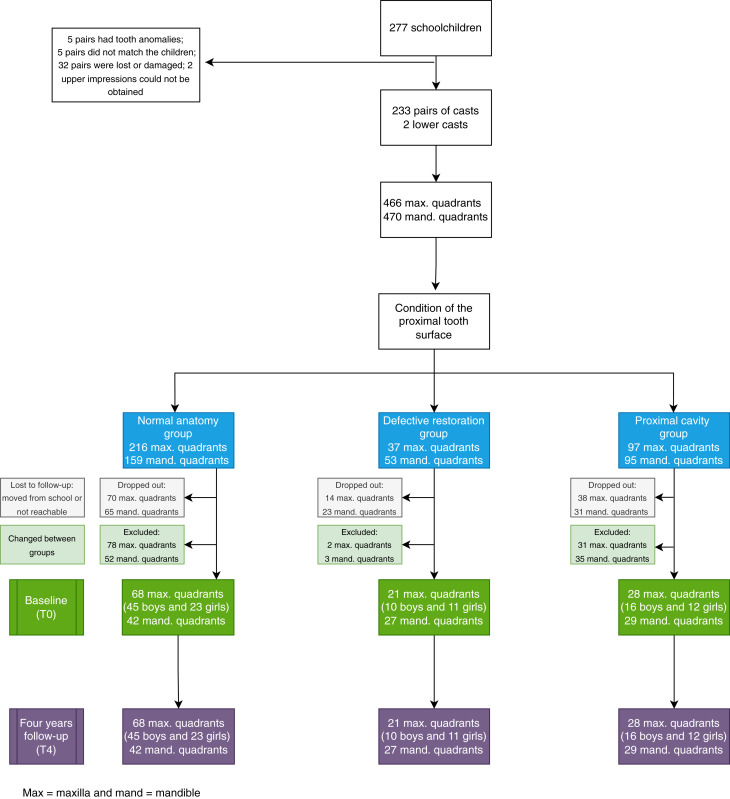
Consolidated standards of reporting trials (CONSORT) flow diagram.

The Consort diagram is presented in Fig. 2. From the original sample of 277 children included in the clinical trial, five children were excluded because they had tooth anomalies (supernumerary or missing lateral primary incisor). It was impossible to obtain maxillary impressions of two children because they experienced severe nausea. At baseline, 37 pairs of casts could not be measured as they had been either damaged or lost during transportation, or the casts did not match the respective children. A total of 233 pairs of casts (130 boys and 103 girls) and two mandibular casts were measured at baseline. Every child that remained in the cohort study had the mouth split in four corresponding quadrants and these were assessed independently. Quadrants were excluded if the children were not followed up for 4 years, if new proximal cavities in the primary molars were present or when premature primary molar loss occurred, and if restorations failed after 2 years.

### Method error

The kappa-coefficient for the inter-examiner agreement for assessing the status of the proximal tooth surfaces was 0.80, which indicated substantial agreement between the two examiners.

The intra-examiner agreement is presented in Table 1. Pearson’s correlation coefficient for the intra-examiner agreement ranged from moderate (0.67 for tooth displacement) to very high (0.99 for D+E space). There was a statistically significant difference between first and second measurement for tooth displacement, but the difference was small (mean diff = 0.14 mm).

**Table 1:** Intra-observer agreement (Pearson’s correlation coefficient, mean difference, 95% confidence interval and *p* value) of the D+E space in the maxilla and the mandible, and displacement of the premolars (in mm).

Variable	Correlation	Mean difference	95% CI of mean dif	*p*
D+E space in the maxilla	0.997	0.01	[−0.04 to 0.05]	0.703
D+E space in the mandible	0.994	0.01	[−0.04 to 0.05]	0.993
Displacement of the premolar	0.672	0.14	[−0.06 to 0.34]	<0.001

**Note:**

Paired sample correlation test.

### D+E space in the maxillary and mandibular dental arch

The mixed model ANCOVA results revealed a statistically significant difference between groups (maxilla *p* = 0.001; mandible *p* < 0.001) and between evaluation times (*p* < 0.001), for the D+E space for both the maxilla and mandible. A sex difference related to the D+E space for the maxilla was found (*p* = 0.007) and therefore the results regarding the maxilla are presented separately for boys and girls.

**Figure 3:**
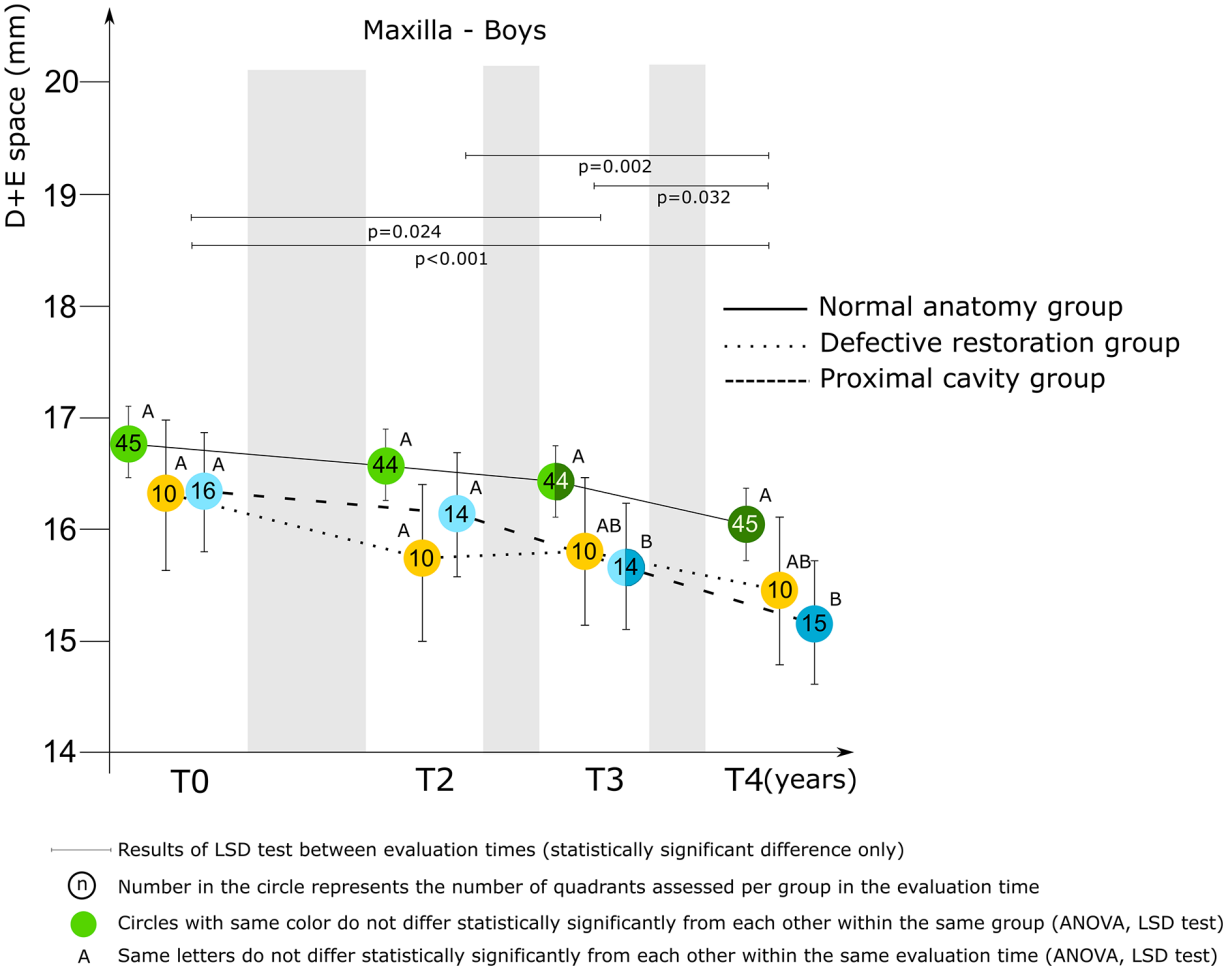
Mixed model ANCOVA results of the D+E space (mean score and 95% CI) for the three groups by evaluation time for boys in the maxilla. Significant difference between groups at each evaluation time are denoted by different letters (A to C).

**Figure 4:**
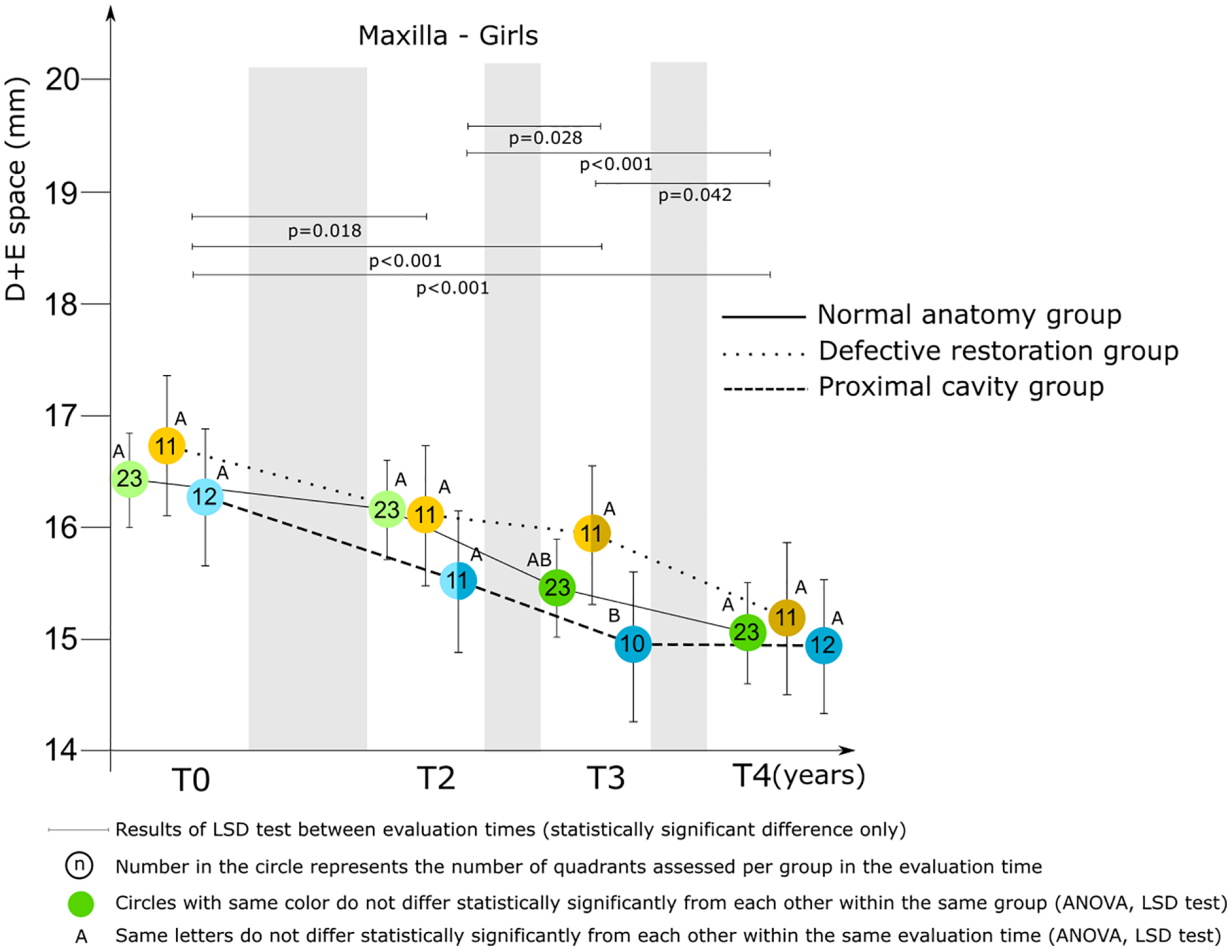
Mixed model ANCOVA results of the D+E space (mean score and 95% CI) for the three groups by evaluation time for girls in the maxilla. Significant difference between groups at each evaluation time are denoted by different letters (A to C).

*Maxilla*. Figure 3 presents the relationships between group and D+E space by evaluation time for boys. The relationships for girls are presented in Fig. 4. There was no statistically significant difference between groups for the D+E space for boys at baseline, and T2. At T3 and T4 the difference was statistically significant. Boys belonging to the proximal cavity group had a shorter D+E space (T3: 15.66 ± 0.98 mm; T4: 15.18 ± 0.97 mm) than boys belonging to the normal anatomy group (T3: 16.40 ± 1.08 mm; T4: 16.01 ± 1.23 mm). For girls there was a statistically significant difference for the D+E space between the normal anatomy group and the proximal cavity group at T3 only.

**Figure 5:**
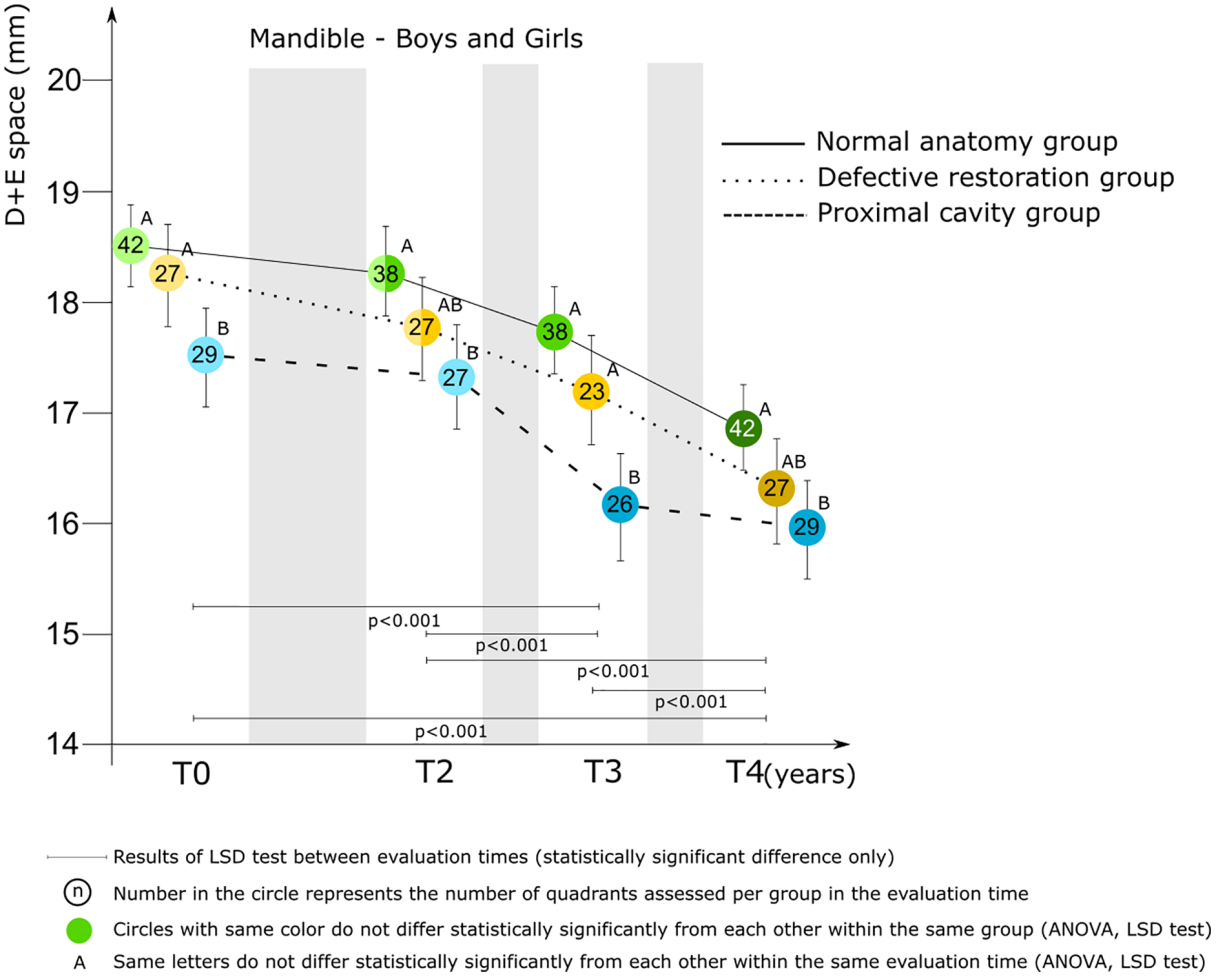
Mixed model ANCOVA results of the D+E space (mean score and 95% CI) for the three groups by evaluation time for boys and girls combined in the mandible. Significant difference between groups at each evaluation time are denoted by different letters (A to C).

*Mandible*. Figure 5 presents the relationships between group and D+E space by evaluation time for the mandible of boys and girls combined. At all evaluation points, the D+E space was statistically significantly shorter for children belonging to the proximal cavity group compared to those of the normal anatomy group. At T4 the mean score was 15.94 ± 1.07 mm (proximal cavity group) and 16.86 ± 1.41 mm (normal anatomy group).

There was no significant difference between the D+E space in quadrants with defective restorations and those with normal anatomy in the mandible and in the maxilla.

### Tooth displacement

The mean score and 95% confidence interval of tooth displacement by group is presented in Table 2. No significant difference among groups in the maxilla (*p* = 0.948) and mandible (*p* = 0.057) was observed. The mixed model ANCOVA results showed that sex was associated to tooth displacement in the maxilla (*p* < 0.001) (Table 3). Girls had larger tooth displacement scores (mean score: 2.11 ± 0.35 mm) when compared to boys (mean score: 1.38 ± 0.31 mm). In the mandible, no association was found (Table 3).

**Table 2:** Descriptive statistics (mean, 95% CI, and *p*) of displacement in mms of the premolars in quadrants with normal anatomy, defective restoration and proximal cavity in both arches.

	Maxilla				Mandible			
Group	Mean	95% CI	*n*	*p*	Mean	95% CI	*n*	*p*
Normal anatomy	1.78	[1.54–2.02]	69	0.948	1.66	[1.40–1.92]	42	0.057
Defective restoration	1.70	[1.28–2.11]	21		1.24	[0.90–1.59]	27	
Proximal Cavity	1.76	[1.40–2.11]	28		1.22	[0.90–1.53]	29	

**Note:**

*n*, Number of quadrants assessed.

**Table 3:** Influence of group, age, and sex on the displacement of the premolars in the maxilla and mandible (mixed model ANCOVA).

	Maxilla			Mandible		
Variable	F	df	*p*	F	df	*p*
Group	0.053	2	0.948	2.965	2	0.057
Age	0.101	1	0.751	0.604	1	0.439
Sex	13.726	1	<0.001	0.037	1	0.847
Group * sex	0.929	2	0.398	0.172	2	0.842

### Tooth impaction

No differences between groups were found (chi-square test) for impaction of the premolars neither in the maxilla nor in the mandible (Table 4).

**Table 4:** Impaction of the premolars. Cross-tabulation associating groups (normal anatomy, defective restoration, and proximal cavity group) and number of quadrants with premolar impaction.

	Maxilla			Mandible		
Group	Impacted	No impaction	*p*	Impacted	No impaction	*p*
Normal anatomy	5	64	0.921	1	41	0.302
Defective restoration	1	20		1	26	
Proximal cavity	2	26		3	26	

## Discussion

### Methodology

This sub-study was nested in a clinical trial and relied for its sample size on that of the main trial (Mijan et al., 2014). As no other study had investigated the effect of the status of the proximal tooth surface on the intra-arch variables, no basic information was available to estimate the optimal sample size for such a study. In addition, attrition of the sample over the 4-year period was substantial due to loss-to-follow-up or exclusion of quadrants, which reduced the power of the study. In the fourth year many children left the primary school for a school with a higher level of education. Intense efforts were made to trace these children, but many could not be recalled. Exclusion was mainly due to quadrants in which the status of the approximal surfaces of the primary molars changed; for example, quadrants with primary molars that had belonged to the normal anatomy group at baseline but developed a proximal cavity, received a restoration, or became prematurely lost in the years following. To avoid differences between groups due to the large number of loss-to-follow-up, only quadrants that were followed up for 4 years were included in the study. Therefore, the results of the current study provide the basis for calculating an adequate sample size for future studies on this topic in an environment such as the current one.

The number of dental casts in the defective restorations and the proximal cavity groups proved to be lower than the number of casts in the normal anatomy group. The normal anatomy group was composed of two conditions, a true sound proximal primary molar surface and a proximal primary molar surface cavity restored with ART/HVGIC or amalgam with proximal contact to an adjacent tooth. Combining these two surface conditions was permitted because no statistically significant difference existed between these two surface conditions and the intra-arch orthodontic variables tested. The group with defective restorations reflects a common situation in pediatric dental care as many defective restorations in primary molars are not re-restored, particularly at this age with the exfoliation time approaching and the substantial percentage of primary molars with defective restorations that exfoliate without symptoms (Hilgert et al., 2016). The number of casts in the proximal cavity group was lower due to the loss of casts in the UCT-group, that consisted predominantly of open proximal cavities, during transportation at the start of the study.

The mixed model ANCOVA and the LSD test that follow it are common statistical tests for a study such as the present one. There is, therefore, no reason to doubt the outcomes of the present study as performed under the prevailing conditions. Nevertheless, a larger sample size, particularly for the defective restorations and proximal cavity groups, would have been an asset and should be strived for in future investigations on this topic.

Measuring variables accurately is essential for constructing a reliable database. Outcomes of reproducibility tests showed that the calculation of the D+E spaces and the determination of the components of the status of the proximal tooth surface were performed at a high level of accuracy.

### Main findings

The null hypothesis was partially accepted. There is neither a difference in the mean scores of impaction and displacement of the premolars in both arches between groups nor for the D+E space in quadrants with defective restorations and those with normal anatomy in the mandible and in the maxilla. But there were significant differences for the D+E space between quadrants with proximal cavity and those with normal anatomy in the mandible and in the maxilla for boys after 3 and 4 years, and for girls after 3 years. This is the first study to investigate the effect of defective restorations and left ‘open’ proximal cavities in primary molars on intra-arch space and on malocclusion. It means that, in addition to the absence of a significant difference in exfoliation of symptom-free primary molars between restored and ‘open’ proximal cavities as earlier reported (Mijan et al., 2014), there is now also absence of proof that keeping proximal cavities open and cleaning them regularly with toothbrush and toothpaste causes tooth displacement and tooth impaction of premolars at age 10–11 years. It is also worth mentioning that the outcome is alike for both boys and girls. The only difference was that girls had no difference between groups while boys showed a statistical difference for the D+E space between normal anatomy quadrants and proximal cavity quadrants. However, this difference might not have important clinical implications since no difference in tooth displacement and impaction was observed. In addition, the present study provided proof that re-restoring defective restorations in primary molars is not necessary for preventing space loss for the permanent successors, which is an important finding in relation to reduction of the burden of dental care and costs.

The study findings may have major implications for the manner tooth cavities in primary molars can be treated in future. While restoring cavities with amalgam or resin composite was the only treatment available some two decades ago, these days tooth cavities can be treated successfully through applying silver diamine fluoride (SDF) (Seifo et al., 2019), placing a Hall-crown (Innes et al., 2017), restoring with ART/HVGIC (Frencken, Liang & Zhang, 2021), and brushing them plaque-free as part of the UCT method. Because the UCT method investigated the use of hand instruments for increasing access to initially insufficiently wide proximal cavities, this treatment can be carried out in countries with meagre financial resources and the consequent limited dental equipment. It is also advocated for use in well-resourced countries because it reduces dental anxiety among children compared to the treatment with the bur, but enlarging of initially insufficiently wide proximal cavities can, off course also be performed with the bur (Santamaria et al., 2015).

These more recently introduced treatments are considered child-friendly and may increase access to dental care for child populations if professional education, knowledge, and adequate dental products are available among the dental profession (Banerjee et al., 2017; Innes et al., 2017; Seifo et al., 2019; Frencken, Liang & Zhang, 2021). Their application also offers the opportunity to operate in tandem with parents/caregivers, which may lead them to a much better understanding of how to keep the primary and, particularly, the permanent dentition free from carious lesions. This may prevent extraction of primary molars at an early age. Overall, the outcome of the present study shows that increased need for orthodontic treatment is not to be expected, while cleaning open proximal cavities in primary molars with toothpaste and toothbrush may contribute to a reduction in healthcare costs for governments and patients.

The total loss of space in the maxilla and mandible was relatively small at the end of the follow-up period (T4), and tooth displacement and tooth impaction of premolars among the groups were comparable. No other study was found that associated tooth impaction and tooth displacement with cavitated dentine carious lesions, but some cross-sectional studies associated the increase of crowding, asymmetry of antero-posterior canine relationship, and lower midline deviation with cavitated dentine carious lesions (Ben-Bassat, Harari & Brin, 1997; Caplin, Evans & Begole, 2015). In a 6-year follow-up study, Northway and Wainright (Northway & Wainright, 1980) reported that large open cavities were associated with a decrease in the D+E space of between 0.5 and 1.0 mm, but the difference was only statistically significant for 9-year-old children in the maxilla and 10-year-olds in the mandible. The authors suggested that this difference occurred at this age, because cavitated primary molars exfoliated 1 year earlier than carious lesion-free primary molars (Northway & Wainright, 1980). This finding was also observed in the main clinical trial (Mijan et al., 2014).

Future studies with larger sample sizes should be designed to confirm our findings and to expand the analysis to the vertical and transverse dimensions.

## Conclusions

Compared to quadrants with a normal anatomy those with open proximal cavities of the primary molars showed a tendency to a shorter D+E space in the mandible over a 4-year follow-up period. The same was applicable for quadrants in the maxilla for boys after 3 and 4 years, and for girls after 3 years. However, the shorter D+E space had no negative implication regarding impaction and displacement of the successor teeth. Primary molars with open proximal cavities that are cleaned with toothbrush and toothpaste do not result in displacement and impaction of the successor teeth, neither do primary molars with defective restorations in proximal tooth surfaces.

## Supplemental Information

10.7717/peerj.14008/supp-1 Supplemental Information 1 Raw data. All the schoolchildren included in the study and the groups that they belong to it.

## Additional Information and Declarations

### Data Availability

The following information was supplied regarding data availability: The raw data are available in the Supplemental File.
